# Single-cell hdWGCNA reveals metastatic protective macrophages and development of deep learning model in uveal melanoma

**DOI:** 10.1186/s12967-024-05421-2

**Published:** 2024-07-29

**Authors:** Yifang Sun, Jian Wu, Qian Zhang, Pengzhen Wang, Jinglin Zhang, Yonggang Yuan

**Affiliations:** 1 Department of Ophthalmology, Guangzhou Red Cross Hospital of Jinan University, Guangzhou, Guangdong 510220 China; 2 Department of Otorhinolaryngology-Head and Neck Surgery, Guangzhou Red Cross Hospital of Jinan University, Guangzhou, Guangdong 510220 China; 3 Guangzhou Institute of Traumatic Surgery, Guangzhou Red Cross Hospital of Jinan University, Guangdong, 510220 China; 4 Guangzhou Aier Eye Hospital, Jinan university, Guangzhou, 510000 China

**Keywords:** Uveal melanoma, Macrophage, Metastatic, Prognosis

## Abstract

**Background:**

Although there has been some progress in the treatment of primary uveal melanoma (UVM), distant metastasis remains the leading cause of death in patients. Monitoring, staging, and treatment of metastatic disease have not yet reached consensus. Although more than half of metastatic tumors (62%) are diagnosed within five years after primary tumor treatment, the remainder are only detected in the following 25 years. The mechanisms of UVM metastasis and its impact on prognosis are not yet fully understood.

**Methods:**

scRNA-seq data of UVM samples were obtained and processed, followed by cell type identification and characterization of macrophage subpopulations. High-dimensional weighted gene co-expression network analysis (HdWGCNA) was performed to identify key gene modules associated with metastatic protective macrophages (MPMφ) in primary samples, and functional analyses were conducted. Non-negative matrix factorization (NMF) clustering and immune cell infiltration analyses were performed using the MPMφ gene signatures. Machine learning models were developed using the identified metastatic protective macrophages related genes (MPMRGs) to distinguish primary from metastatic patients. A deep learning convolutional neural network (CNN) model was constructed based on MPMRGs and cell type associations. Lastly, a prognostic model was established using the MPMRGs and validated in independent cohorts.

**Results:**

Single-cell RNA-seq analysis revealed a unique immune microenvironment landscape in primary samples compared to metastatic samples, with an enrichment of macrophage cells. Using HdWGCNA, MPMφ and marker genes were identified. Functional analysis showed an enrichment of genes related to antigen processing progress and immune response. Machine learning and deep learning models based on key genes showed significant effectiveness in distinguishing between primary and metastatic patients. The prognostic model based on key genes demonstrated substantial predictive value for the survival of UVM patients.

**Conclusion:**

Our study identified key macrophage subpopulations related to metastatic samples, which have a profound impact on shaping the tumor immune microenvironment. A prognostic model based on macrophage cell genes can be used to predict the prognosis of UVM patients.

**Supplementary Information:**

The online version contains supplementary material available at 10.1186/s12967-024-05421-2.

## Instroduction

Uveal melanoma (UVM) is currently the most common primary adult eye tumor, with incidence rates varying significantly across age, race, and latitude [1]. An analysis of the Surveillance, Epidemiology, and End Results (SEER) Project database from the National Cancer Institute, spanning a 36-year period from 1973 to 2008 (including 4,070 cases of primary UVM), showed an age-adjusted incidence rate of 5.1 cases per year per 100,000 for UVM [2]. UVM is more common in older adults, with an age-specific incidence rate that gradually increases and peaks at around 70 years of age, and then stabilizes after 75 years of age [3]. UVM is more common in individuals of white racial background. The estimated ratio of UVM incidence between black and white individuals is approximately 1:15 to 1:50 [4, 5]. Using data from 1,352 patients between 1992 and 2000 from the SEER project, the relative risk (RR) of UVM was calculated for different racial groups (black, Asian, Pacific Islander, Hispanic, and non-Hispanic white). The results showed a ratio of black: Asian: Hispanic: non-Hispanic white of 1:1.2:5:19 [6].

Approximately half of patients diagnosed with primary UVM will develop metastatic uveal melanoma. Although effective treatments (plaque brachytherapy, proton beam therapy, and enucleation surgery) exist to eradicate and prevent local recurrence of intraocular UVM, there is currently no effective treatment for metastatic UVM [7]. Despite aggressive treatment options such as radiation or enucleation for primary UVM, approximately 50% of patients still experience metastasis [8]. This primary disease phenotype is related to specific gene expression patterns. Uveal melanoma metastasis most commonly occurs in the liver and is believed to be driven by high levels of growth factors (such as epidermal growth factor, insulin-like growth factor, and hepatic growth factor) [9]. The clinical presentation of UVM can be broadly classified into two categories: (a) UVM diagnosed and limited to the local eye, and (b) UVM that develops metastasis and ultimately leads to death due to distant disease. UVM with chromosome 3 loss has the worst prognosis, while UVM with chromosome 6p amplification has the best prognosis [10, 11].

Macrophages are an important component of the immune system and play a crucial role in tumor immunity [12]. Traditionally, macrophages that express high levels of tumor necrosis factor (TNF), inducible nitric oxide synthase (iNOS), or MHC Class II molecules are considered to have anti-tumor effects. On the other hand, macrophages that express high levels of arginase-1 (ARG1), IL-10, CD163, CD204, or CD206 are considered to have pro-tumor effects [13]. Recently, the rapid development of scRNA-seq technology has provided a powerful tool for revealing the heterogeneity of tumor-infiltrating immune cells [14]. In this study, we used single-cell high-dimensional weighted gene co-expression network analysis (HdWGCNA) to identify key genes associated with metastatic protective macrophage subpopulations by comparing primary and metastatic sites of UVM and explored the potential molecular mechanisms involved. We also built machine learning and deep learning models based on macrophages related genes (MPMRGs) to distinguish primary and metastatic patients which can benefit clinical diagnosis. A prognostic signature with MPMRGs was also established. Our research aims to provide new treatment targets and strategies for improving the outcome and prognosis of UVM patients.

## Methods

### Transcriptome Data Download and Processing

The “GEOquery” R package was used to download the UVM datasets GSE22138 (including 28 primary samples and 35 metastatic samples) and GSE58294 (including 18 primary samples and 11 metastatic samples) as the training and validation cohorts for machine learning and deep learning, respectively, and GSE84976 as the validation cohort for constructing the prognostic model. Additionally, the “GDC TCGA Uveal melanoma (UVM)” data were downloaded from the UCSC Xena database as the training cohort for constructing the prognostic model. The counts data of UVM were converted to TPM data type, and all data were log2 transformed for subsequent analysis.

### Single-cell RNA-seq analysis of UVM samples

The scRNA-seq data analyzed was from Michael A Durante et al. [15]. The UVM single-cell dataset GSE139829 was downloaded from the GEO database (http://www.ncbi.nlm.nih.gov/geo/). After retaining the primary non-metastasized samples and the samples from distal post-metastasized tissues, resulting in 9 samples, data quality control and cell filtering were performed using the “Seurat” package [16]. PCA, UMAP [17], and cell type labeling were applied. Subclustering analysis of the Monocytes and macrophages cluster, cell proportion calculation, pseudo-temporal trajectory analysis using “monocle”, and cell-cell communication analysis using “Cellchat” were conducted [18, 19]. Differential expression analysis and GSEA enrichment were performed on Monocytes and macrophages cells genes.

### HdWGCNA analysis

High dimentional weighted gene co-expression network analysis (hdWGCNA) [20]was performed to identify key genes associated with monocytes and macrophages cells in the metastatic samples. Monocytes and macrophages cell populations were selected from scRNA data, and gene expression correlation matrix, weighted gene co-expression networks, and module detection were conducted. Module-trait relationship analysis identified modules significantly associated with the metastasis group, and hub genes within significant modules were identified based on their intra-module connectivity. The top 80 hub genes were considered the key genes associated with Metastatic protective macrophages (MPMφ) in the primary samples.

### Analysis of Gene Function and Gene Network of 80 MPMφ Genes

The STRING database (https://string-db.org) was used to predict the interaction networks of 80 MPMφ genes, and the optimal confidence level the interactions were determined based on the PPI network is 0.4. We then processed the analyses using Cytoscape software. Enrichment analysis of inflammation response-related macrophage cells genes was performed using Matescape (http://metascape.org/) to screen for biological functions and signaling pathways at *p* < 0.01 and involving at least 3 genes in the provided gene list.

### Sample clustering using non-negative matrix factorization algorithm

Non-negative matrix factorization (NMF) was used to classify patients into different subtypes. The top 50 MPMφ genes obtained by hdWGCNA were used as potential prognostic DEGs. Samples were clustered using the “brunet” method in the “NMF” package of R software, and the optimal number of clusters was determined. Consensus heatmaps were generated, and the distributions of different subtypes were observed. GSVA scoring, chromosome 3 status proportions, and immune cell infiltration estimation using the MCPcounter algorithm from the “IOBR” package were performed across different clusters [21].

### Screening of biomarkers using machine learning techniques

The Least Absolute Shrinkage and Selection Operator (LASSO) regularized regression algorithm from the “glm net” package was used on the top 80 MPMφ genes to determine 5 MPMRGs associated with prognosis. Seven machine learning methods (Logistic Regression, LDA, SVM, Naive Bayes, KNN, rpart, Ranger) were trained on GSE22138 to discriminate between primary and metastatic cancers [22–28], and their ROCs were compared. The Support Vector Machine algorithm (SVM) was used for validation on GSE58294.

### Artificial neural network construction

A Convolutional Neural Network (CNN) model based on mcp/gene scores was constructed for the test cohort GSE22138 using the “keras” package in R software. The predictive performance of the validation cohort GSE58294 was evaluated using the receiver operating characteristic curve (ROC) [29].

### Prognostic model construction and validation

The 5 MPMRGs selected from LASSO regression were analyzed in a multifactorial Cox regression analysis of 80 patients with TCGA data, and a prognostic model was constructed according to the “predict” algorithm in the R package “survMisc”. Patients were categorized into high and low scoring groups based on the median risk score. Survival curves, risk plots, and time-dependent ROC curves were generated to visualize survival differences, patient status, and assess the efficacy of risk scores in predicting OS at 1, 3, and 5 years. The GSE84976 cohort was used as an independent external cohort to validate the prognostic model.

### Statistical analysis

All statistical analyses were conducted using R program (version 4.2.3, https://www.r-project.org). Survival analysis was performed using the Kaplan-Meier method along with the Log-rank test. A p-value < 0.05 was considered statistically significant (*, *p* < 0.05; **, *p* < 0.01; ***, *p* < 0.001).

## Results

### scRNA-Seq and cell typing of primary and metastatic UVM samples

**Fig. 1:**
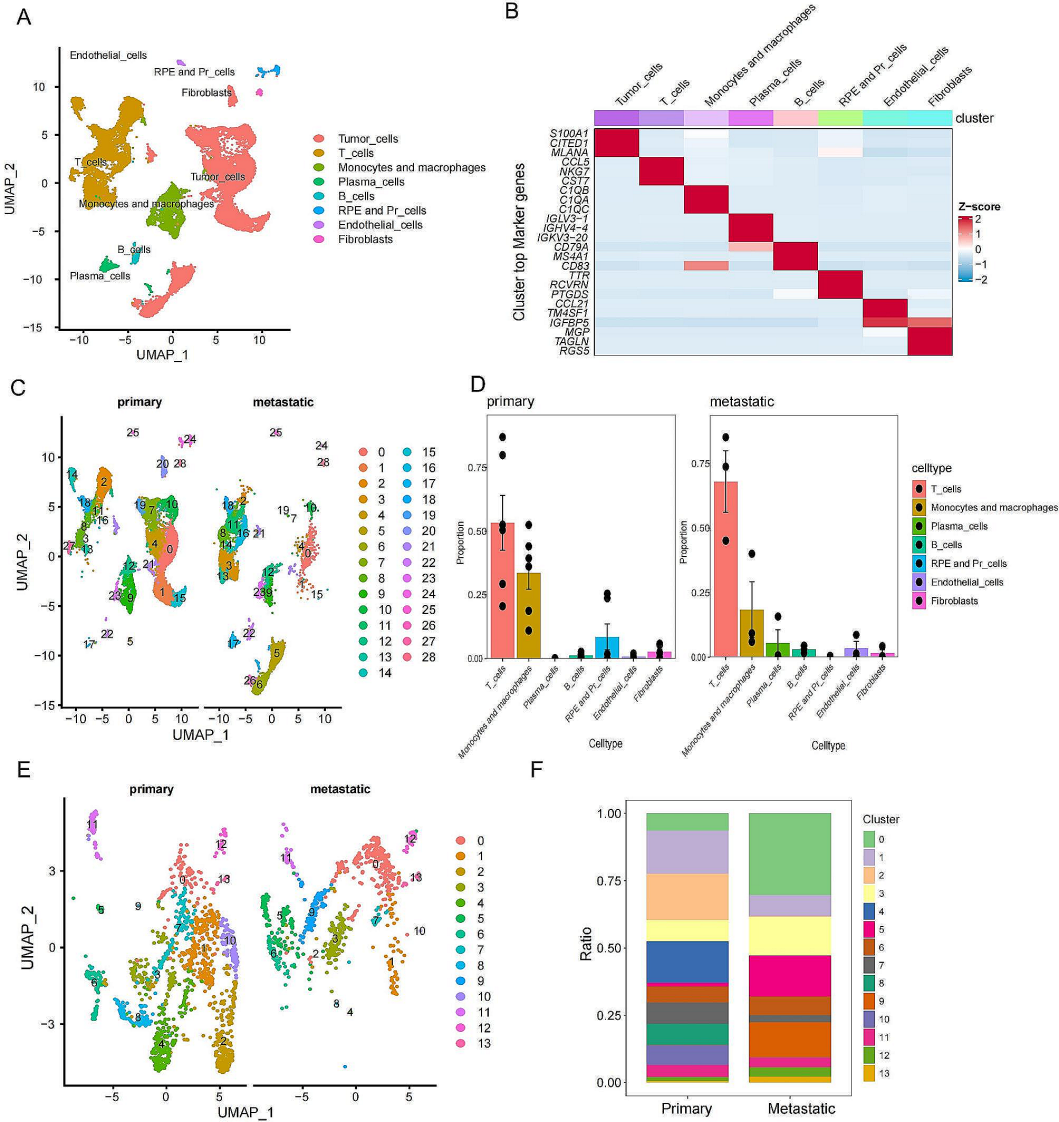
**Single-cell RNA-seq analysis of macrophages in UVM samples.** (**A**) UMAP plots of different subclusters of cells in UVM samples. (**B**) Top marker genes of different subclusters in UVM samples. (**C**) Clustering UMAP plots of different clusters of cells in primary and metastatic lung samples. (**D**) Proportions of different subclusters in primary and metastatic lung samples. (**E**) Clustering UMAP plots of macrophages in primary and metastatic lung samples. (**F**) Proportions of different clusters in macrophages in primary and metastatic lung samples

We obtained a total of 6 primary samples and 3 distal metastasis samples from 10×scRNA-seq of GSE139829. After stringent quality control and data normalization, we obtained transcriptomes for a total of 26,354 cells. PCA and UMAP analysis determined 28 different clusters (Additional file 1: Figure S1A). Then, based on the following cell markers, we performed cell typing: Tumor cells (MLANA, MITF), T cells (CD3D, CD8A), B cells (CD19, MS4A1, CD79A), Plasma cells (CD79A, IGHG1, MZB1), monocytes and macrophages (CD68, CD14), Retinal pigment epithelium and Photoreceptor cells (RCVRN, RPE65), Fibroblasts (COL1A1), Endothelial cells (PECAM1, VWF). These cell markers accurately distinguished these cell types (Additional file 2: Figure S2). We ultimately determined 8 major cell clusters: Tumor cells, T cells, B cells, Plasma cells, Monocytes and Macrophages, RPE and Pr_cells, Fibroblasts, and Endothelial cells. Using the “FindAllMarkers” package, we identified the marker genes for each major cluster and created heatmaps (Fig. 1A and B, Additional file 3: Table S1). Interestingly, we found that monocyte and macrophage cells(cluster 9, 12 ,23) in the metastatic group(12.6%) were significantly lacking compared to the primary group(24.9%) (Fig. 1C and D). To further characterize the heterogeneity of monocyte and macrophage cells, we performed additional clustering analysis. The results showed that monocyte and macrophage cells were divided into 13 subclusters (Additional file 1: Figure S1B). By comparing the UMAP plots and clusters between the two groups, it was clear that there was a significant difference in the number of cells between clusters 2,4,8,10 in the primary group (46.7%) and the metastatic group (0.7%) (Fig. 1E and F).

### Identification and characterization of Metastatic protective macrophages (MPMφ) in the tumor microenvironment

**Fig. 2:**
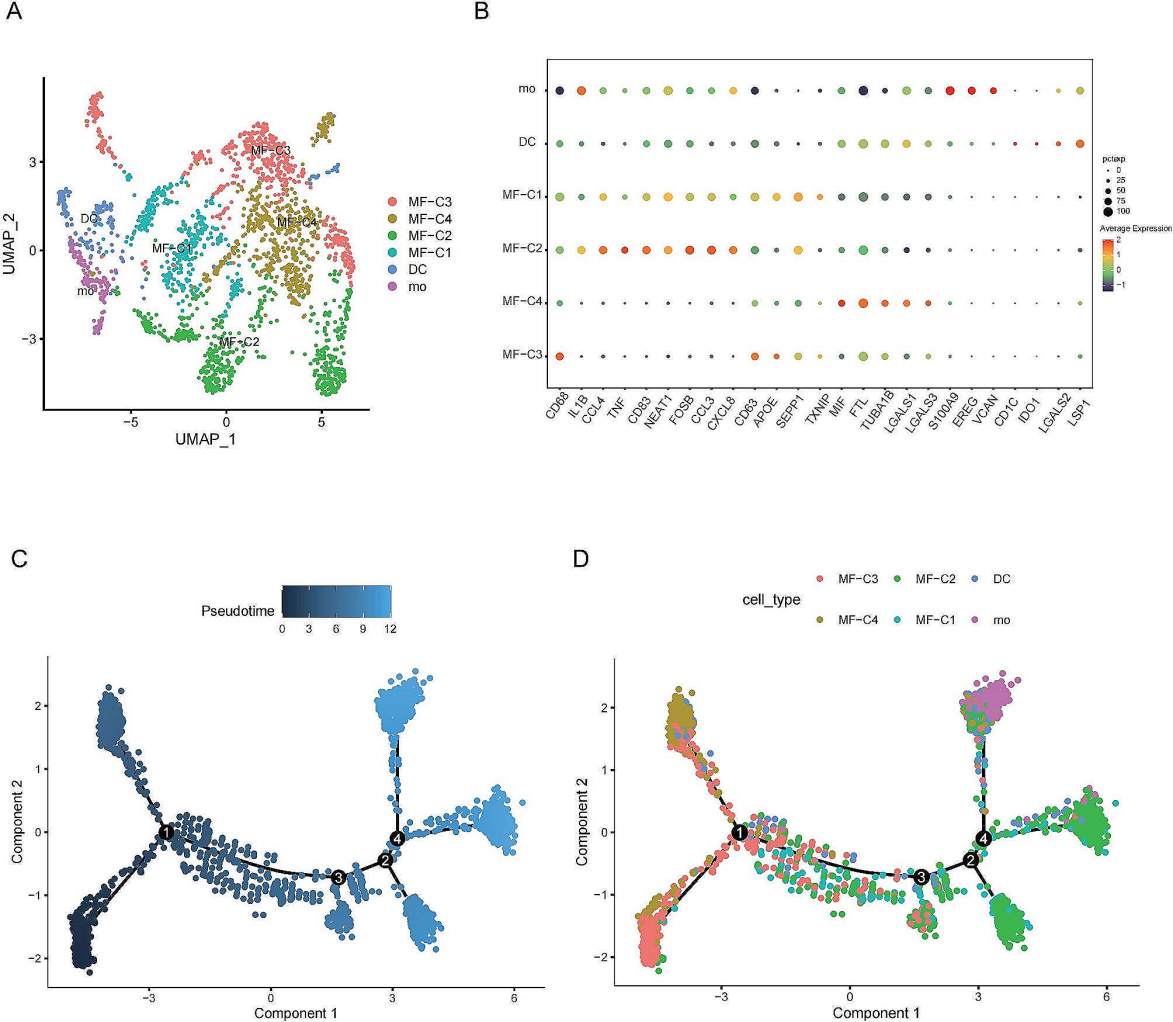
**Macrophage subclustering and Monocle results.** (**A**) Subtyping of macrophage cells. (**B**) Marker genes used to define subtypes of macrophages. (**C**) Monocle time-series plot of macrophage cells. (**D**) Time-series plot of different macrophage subtypes

According to existing literature reports [30], we divided monocytes and macrophages into six categories and listed the main markers for each group, including: monocytes highly expressing monocyte-related genes (such as S100A9, EREG, G0S2, and VCAN); DCs highly expressing CD1C, IDO1, LGALS2, and LSP1; MΦ-C2 highly expressing pro-inflammatory mediators (such as IL1B, CCL3, CXCL8, FOSB, and CD83); MΦ-C3 highly expressing M2-MΦ markers (such as APOE, SEPP1, and TXNIP); MΦ-C1 highly expressing M1/M2-MΦ markers; and MΦ-C4 highly expressing microtubule-associated genes (TUBA1B), ferritin light chain (FTL), and some immunomodulatory molecules (MIF) (Fig. 2A and B, Additional file 1: Figure S1C). We performed a pseudo-temporal analysis using the “Monocle” package on the annotated cells and found that our cells of interest (clusters 2, 4, 8, and 10) were primarily distributed in later-stage differentiation (Fig. 2C-D).

**Fig. 3:**
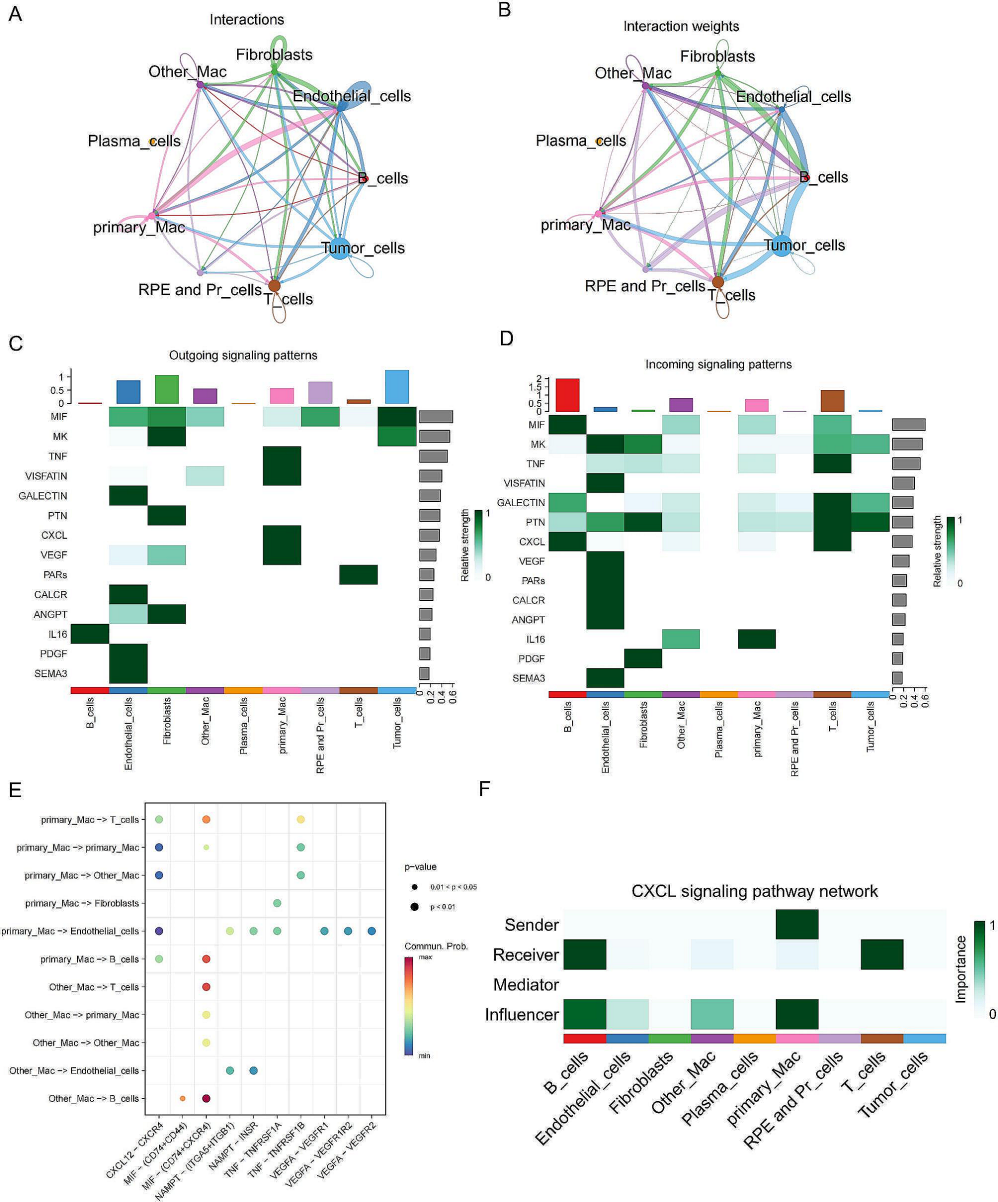
**Comprehensive CellChat analysis of all cell types.** (**A**) Communication interactions network plot for all cell types. (**B**) Communication interaction weights network plot for all cell types. (**C**) Heatmap of signal pathways emitted by all cell types. (**D**) Heatmap of signals received by all cell types. (**E**) Receptor design for primary_Mac and Other_Mac as signal senders. (**F**) The heatmap shows the relative importance of each cell cluster’s centrality measure in the computed CXCL signal network

To differentiate our cells of interest, the “primary_Mac” cluster (clusters 2, 4, 8, and 10), from other cell clusters, we named the other cell clusters “other_Mac.” We then performed cell-cell communication analysis using the “CellChat” package. The communication results showed that monocyte and macrophage cells significantly participated in the MIF, TNF, VISFATIN, CXCL, VEGF, GALECTIN, PTN, and IL16 signaling pathways (Fig. 3C-E). Compared to “other_Mac,” “primary_Mac” significantly participated in the TNF, CXCL, and other inflammatory activation pathways, with active cell-to-cell communication (Fig. 3A-B, Additional file 1: Figure S1D). The pathway heatmap depicted the relative importance of the cells in four different roles: senders, receivers, intermediaries, and influencers. The results showed that primary_Mac played a prominent role as both sender and influencer in the CXCL signaling pathway (Fig. 3F).

### Identification of key gene modules and functional pathways associated with MPMφ cells using high-dimensional weighted gene co-expression network analysis

**Fig. 4:**
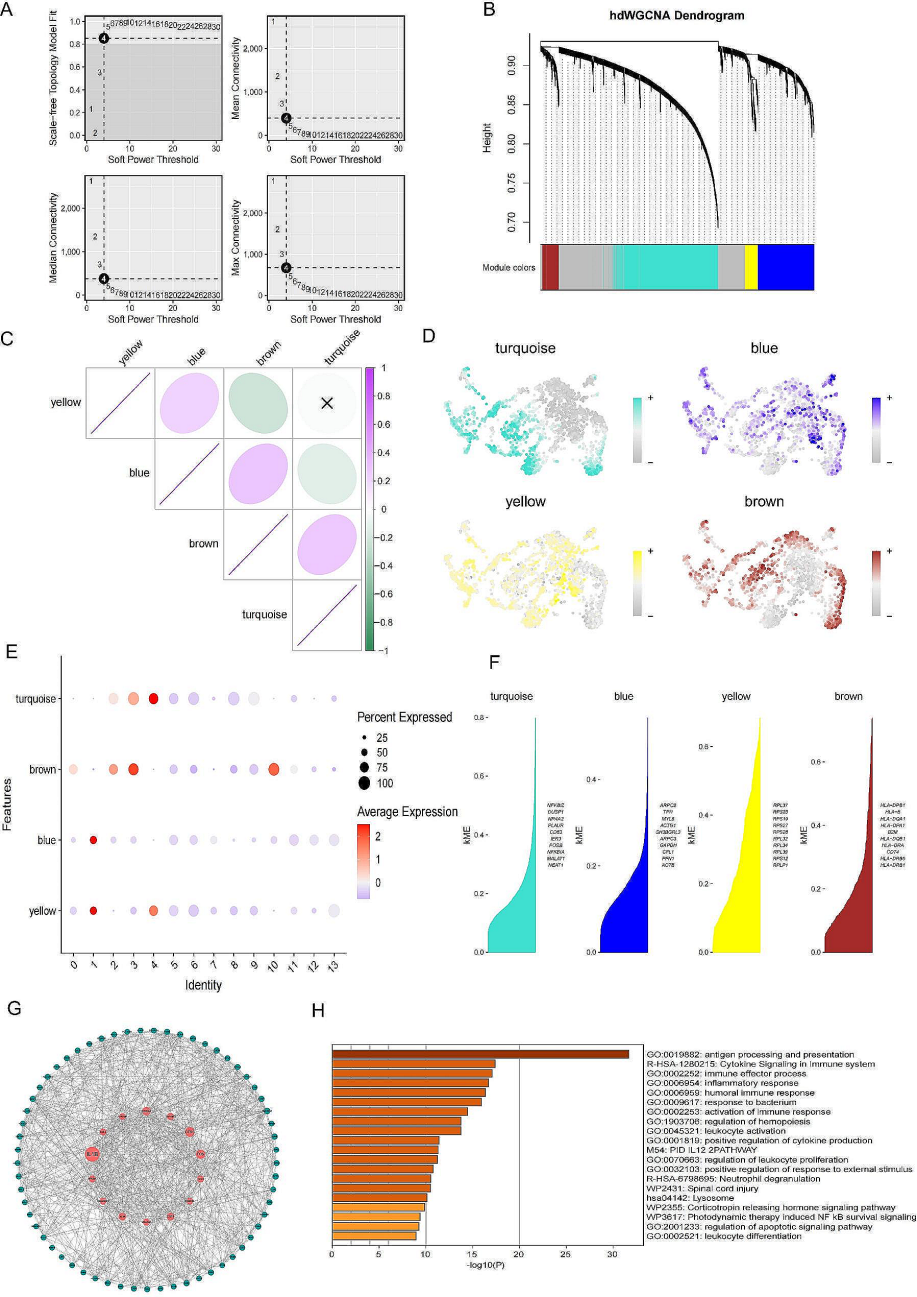
**The WGCNA analysis of macrophage cells showed the key roles of the turquoise module and brown module.** (**A**) Optimal soft threshold selection. (**B**) Construction of co-expression network using the optimal soft threshold of 4, with genes divided into 4 modules and resulting in a dendrogram. (**C**) Correlation analysis between the 4 modules. (**D**) UMAP plot of macrophages with ME staining. (**E**) Module activity for 13 macrophage clusters. (**F**) KMes for each module characterisation gene. (**G**) Gene network of MPMφ cells. (**H**) Barplot of gene enrichment items for MPMφ cells, colored by p-value

We used high-dimensional weighted gene co-expression network analysis (HdWGCNA) to identify the key molecular features of primary macrophages. During the construction of the co-expression network, we observed that when the scale-free topology fitness index reached 0.90, the soft threshold power β was 4 (Fig. 4A) to build an unweighted primary macrophages cell network, achieving the best connectivity. We identified four gene modules (Fig. 4B). The correlation between the four modules is shown in Fig. 4C. Subsequently, we assessed the module scores for the five clusters and found that the turquoise and brown modules were highly activated in clusters 2, 4, 8, and 10 (Fig. 4D, E). And we calculated modular connectivity to determine the connectivity of each gene based on the characterised genes(Fig. 4F). Therefore, we believe that the turquoise and brown modules are related to the primary macrophages cell clusters.

We extracted the top 40 significant genes from the two modules and used them as key genes for primary macrophages cells. We then performed PPI network analysis on the 80 key genes for primary macrophages cells using STRING (Fig. 4G, Additional file 4: Table S2) and conducted enrichment analysis using Metascape (Fig. 4H). In the PPI network, we scored the different genes by the ‘Betweenness’ algorithm, where a larger circle represents a higher score (Fig. 4G, Additional file 5: Table S3). The results showed that these primary macrophages key genes are concentrated in terms related to antigen processing and presentation, immune effector processes, inflammatory response, and activation of immune response, which demonstrated that these macrophages were metastatic protective macrophages (MPMφ).

In addition, with drug data from the cellminer database, we also examined the drug sensitivity of the top 20 important genes in both modules, and we screened for drugs containing more than 10 genes of interest(Additional file 6: Table S4). Ten genes including NFKBIZ, NR4A2, CD74, and PSAP were sensitive to Dabrafenib, 11 genes including CD74, HLA-DRA, CD83, and NR4A2 were sensitive to Hypothemycin, 11 genes including HLA-DRB5, PLAUR, PPP1R15A, DUSP1, and 10 genes sensitive to Simvastatin, 10 genes including HLA-DPA1, PSAP, CD74, NR4A2 sensitive to Vemurafenib. Sixteen genes including HLA-A, PPP1R15A, PSAP, PLAUR were resistant to By-Product of CUDC-305. This reflects the fact that both modules may have some regulatory control over drug resistance and provide some data to guide clinical use.

### Identification of patient subtypes and immune cell infiltration patterns based on MPMφ key genes using NMF clustering and GSEA

**Fig. 5:**
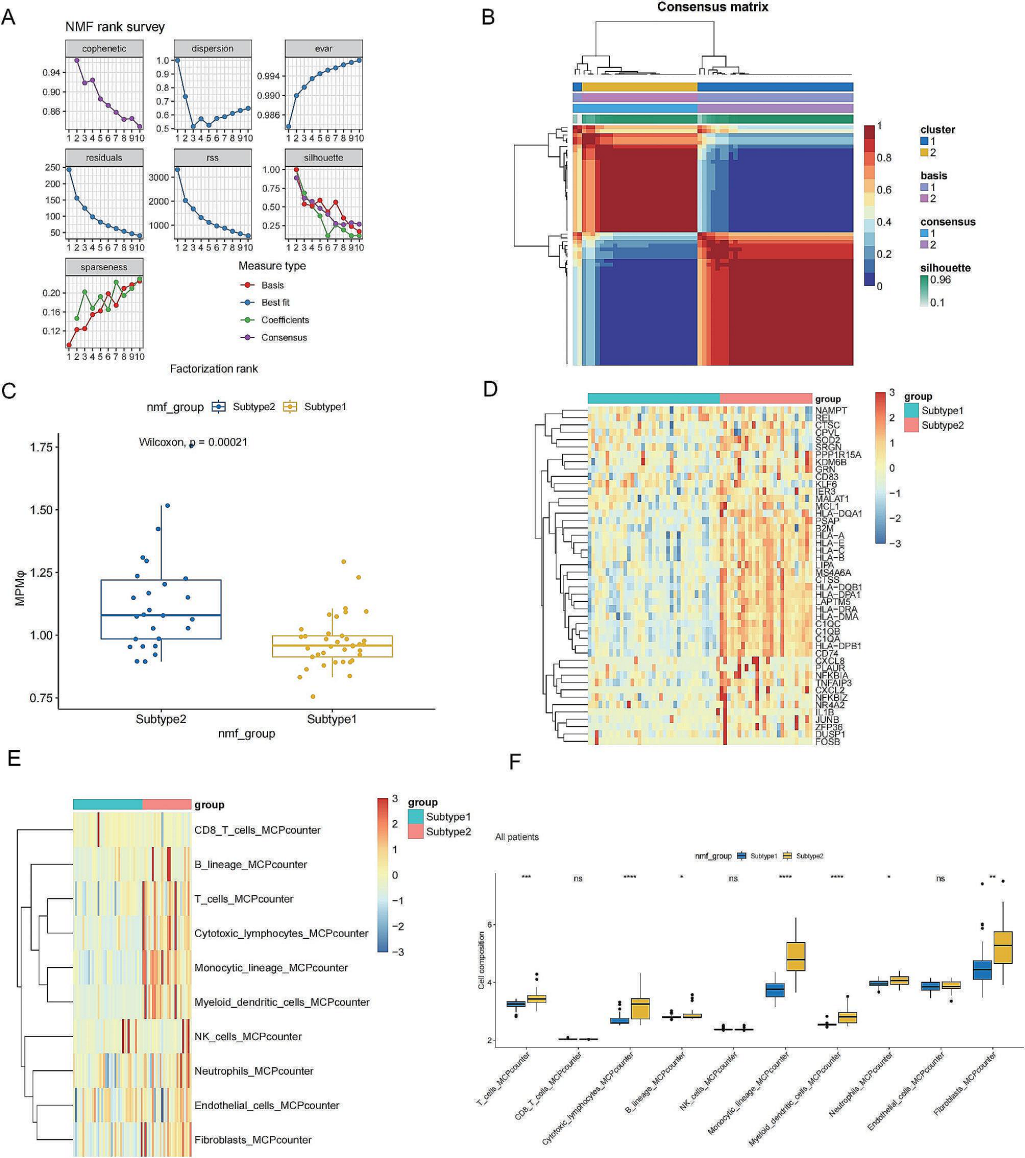
**Identification of MPMφ cell subtypes in UVM.** (**A**) NMF rank survey (**B**) Distinguishing two different subtypes using the NMF algorithm. (**C**) Expression of MPMφ-related genes in the two subtypes. (**D**) Expression heatmap of MPMφ-related genes in the two subtypes. (**E**) Immune infiltration heatmap for the two subtypes. (**F**) Immune infiltration barplot for the two subtypes

We selected the top 25 key genes from each of the two modules identified in the previous analysis and performed non-negative matrix factorization (NMF) clustering on the GSE22138 dataset using the selected 50 MPMφ key genes. Based on the predicted results in Fig. 5A, we selected rank = 2 as the optimal NMF parameter. The NMF clustering classified the patients into two distinct groups: Subtype 1 and Subtype 2 (Fig. 5B). We created a heatmap to compare the expression patterns of the 50 MPMφ genes in the two patient groups. Gene set enrichment analysis (GSEA) using the 50 key genes showed that the expression levels of these genes were significantly higher in Subtype 2 compared to Subtype 1, and this difference was statistically significant (Fig. 5C). The heatmap revealed that most of the genes were highly expressed in Subtype 2 and lowly expressed in Subtype 1 (Fig. 5D).

To estimate the infiltration of immune cells in the different patient clusters, we used the MCPcounter algorithm. The analysis revealed that the levels of T cells, cytotoxic lymphocytes, B lineage cells, monocytic lineage cells, myeloid dendritic cells, neutrophils, and fibroblasts were higher in Cluster 2 compared to Cluster 1 (Fig. 5E, F), indicating patients with high MPMφ processed stronger immune cell infiltration.

### A machine learning model was built using the 5 MPMRGs to differentiate between primary and metastatic UVM patients

**Fig. 6:**
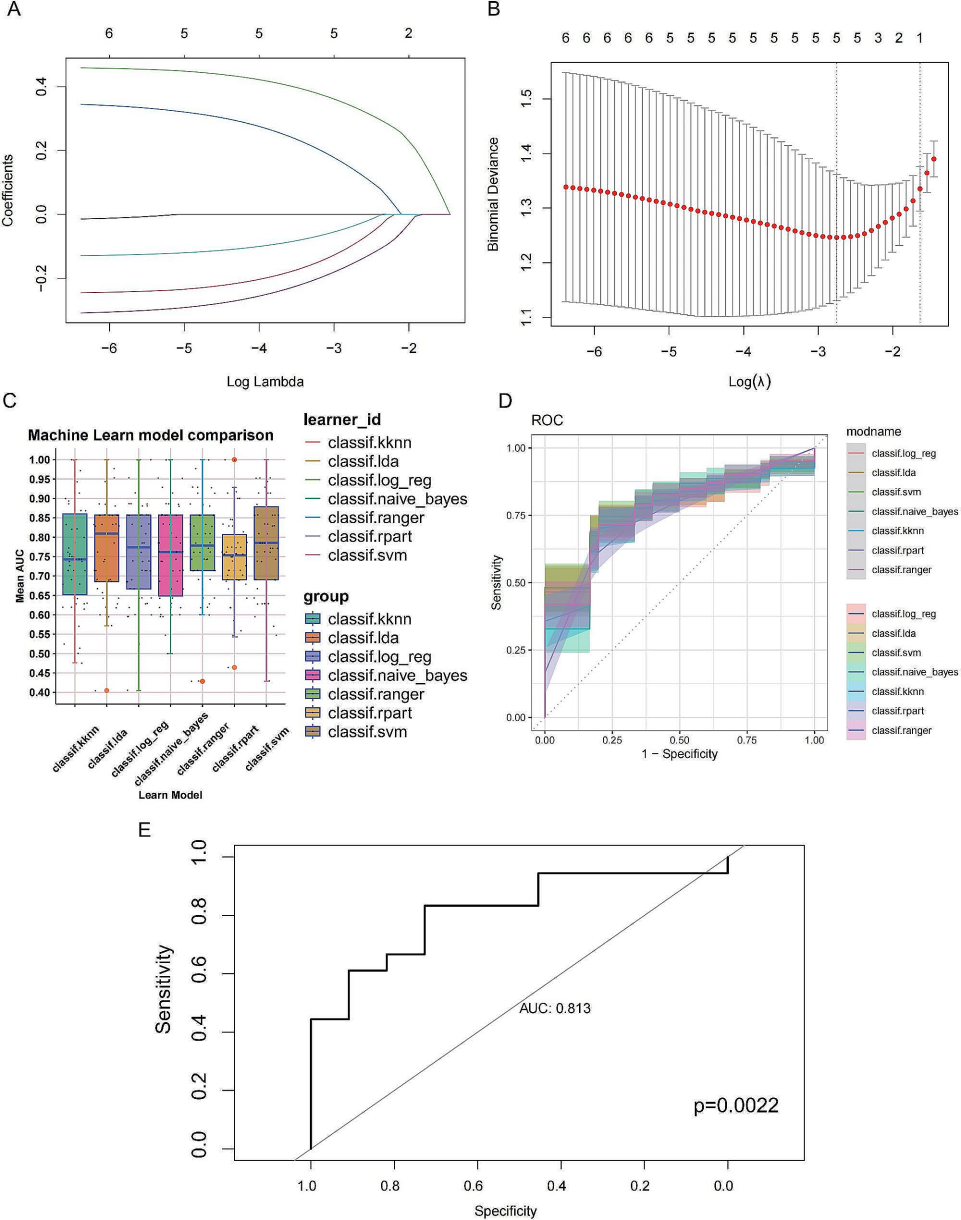
**Developing and validating a machine learning model to distinguish primary patients from metastatic patients.** (**A**, **B**) MPMRGs were identified using LASSO analysis with 5-fold cross-validation. (**C**) AUC comparison of seven machine learning models. (**D**) Area Under the Receiver Operating Characteristic (ROC) curves for seven machine learning models. (**E**) AUC results of the SVM-based model applied to an independent external validation cohort

We selected the top 40 genes from each module, turquoise and brown to identify the signature genes in primary patients from the GSE22138 dataset. Using LASSO regression, we screened for 5 genes (ICAM1, FOS, PDE4B, CPVL, and CD83) (Fig. 6A, B). We then compared the stability of 7 machine learning models (Logistic Regression, LDA, SVM, Naive Bayes, KNN, rpart, and Ranger) to ultimately select the SVM model with the best precision for building the 5 MPMRGs model (Fig. 6C, D). Finally, we used the MPMRGs SVM model to validate another dataset containing 18 primary and 11 metastatic patients from the GSE58294 cohort, showing robust diagnostic performance (Fig. 6E).

### Deep learning CNN model differentiates primary and metastatic patients using MPMRG expression and cell type associations

**Fig. 7:**
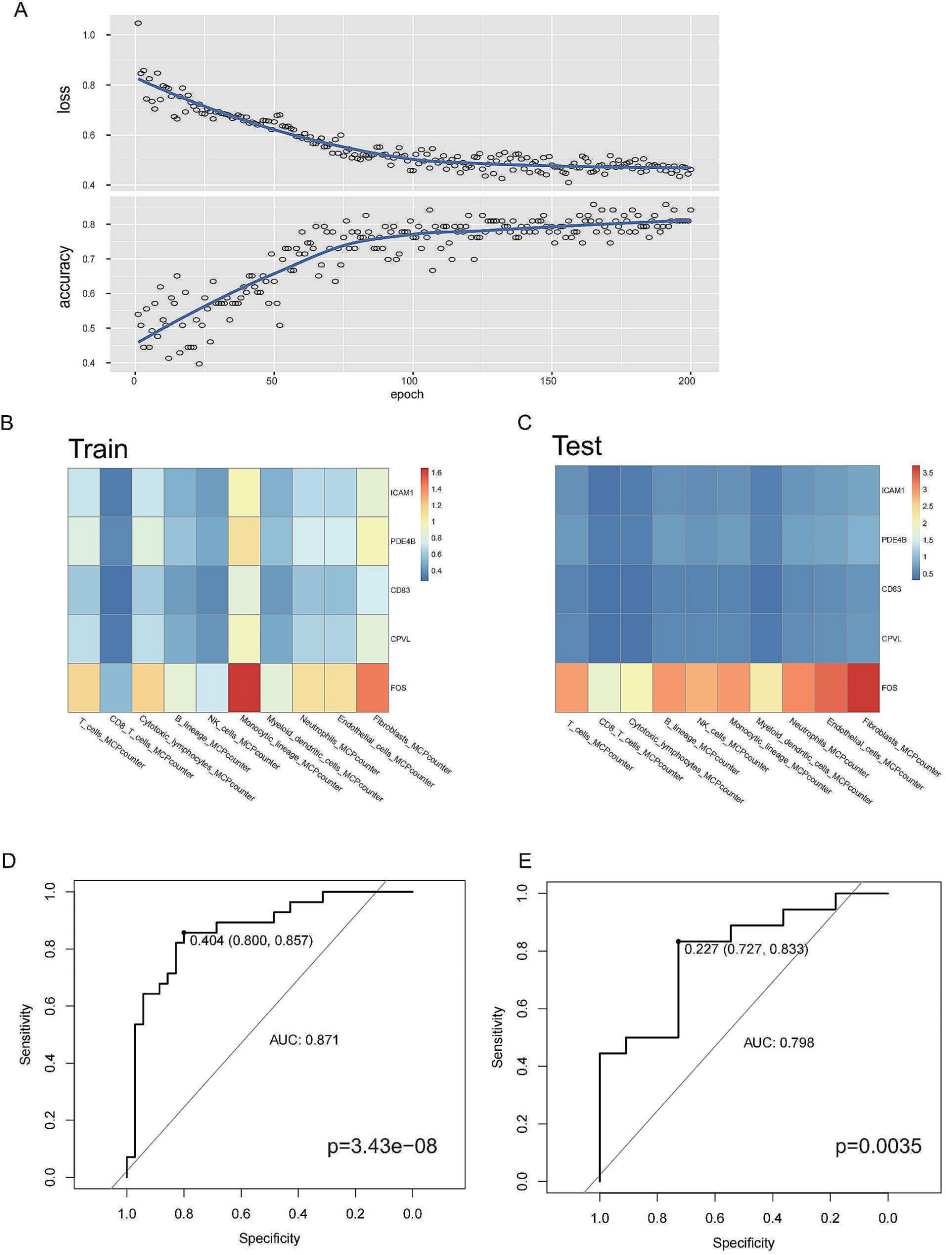
**Creating a deep learning model utilizing artificial convolutional neural networks for the purpose of distinguishing between primary and metastatic patients.** (**A**) Training process of the convolutional neural network. (**B**) Heatmap cards of patients in the training set, made based on mcpcounter scores and MPMRGs expression levels. (**C**) Heatmap cards of patients in the validation set, made based on mcpcounter scores and MPMRGs expression levels. (**D**) AUC for diagnostic performance in the training set. (**E**) AUC for diagnostic performance in the validation set

The GSE22138 cohort was used as the training set, while the GSE58294 cohort served as the validation set. The training was set to 200 iterations to reduce bias and improve accuracy (Fig. 7A). Various cell type scores were calculated using MCPcounter for both cohorts. The expression levels of MPMRGs were then compared with different cell types, including immune cells, fibroblasts, and epithelial cells, for each patient in both the training and validation sets. A two-dimensional array was constructed for each patient using this method to demonstrate the associations between these key genes and specific cell types, and the findings were visualized using heatmaps (Fig. 7B, C).

The receiver operating characteristic (ROC) curves for the primary and metastatic cohorts were 0.871 and 0.798, respectively, indicating that the deep learning CNN model has high sensitivity and accuracy for differentiating primary and metastatic patients (Fig. 7D, E).

### Establishment of a MPMRGs-based prognostic model for UVM

**Fig. 8:**
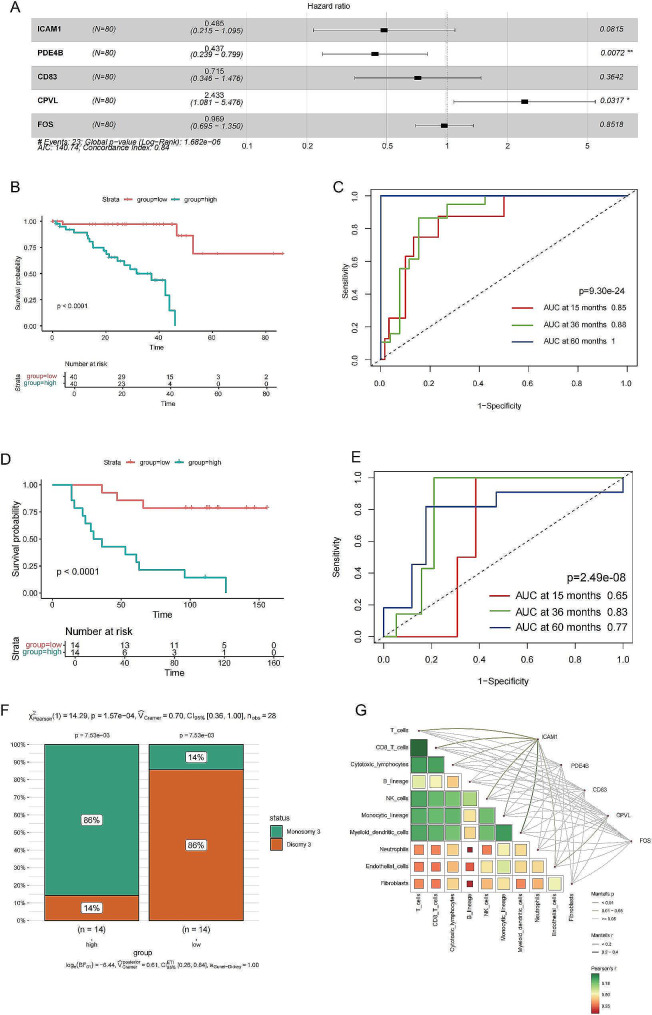
**Construction and validation of risk scoring model based on MPMRGs.** (**A**) Forest plot for the Cox regression model using the TCGA-UVM cohort as the training set. (**B**) Kaplan-Meier curves for the TCGA-UVM cohort. (**C**) Receiver Operating Characteristic (ROC) curve for the TCGA-UVM cohort. (**D**) Kaplan-Meier curves for the GSE84976 cohort. (**E**) Receiver Operating Characteristic (ROC) curve for the GSE84976 cohort. (**F**) Proportion of chromosome 3 states after stratification by risk score median in the GSE84976 cohort, used as the validation set. (**G**) Correlations of 5 MPMRGs with immune cell infiltration

We constructed an MPMRGs-based prognostic model using a cohort of 80 patients from the TCGA dataset as the training group and employed Cox regression analysis to establish the model (Fig. 8A). The survival package’s predicate function was utilized to risk-score the 80 patient samples. Samples were subsequently classified into high-risk and low-risk groups based on the median risk score. Kaplan-Meier survival analysis demonstrated that the high-risk group had a higher mortality rate and shorter overall survival compared to the low-risk group (*p* < 0.001, Fig. 8B, C).

The MPMRGs-related prognostic model was further validated in an independent external cohort of 28 patient samples from the GSE84976 dataset. In this cohort, Kaplan-Meier survival analysis showed that the high-risk group had a higher mortality rate and shorter overall survival than the low-risk group (*P* < 0.001, Fig. 8D, E). Additionally, the model demonstrated independent prognostic value beyond other clinical features. The status of chromosome 3 was crucial for UVM prognosis. Monosomy 3 had a worse prognosis than disomy 3, and the high-risk group had a higher proportion of monosomy 3 (Fig. 8F). Moreover, correlation analysis revealed that 5 MPMRGs had relationships with immune cells, especially ICAM1 (Fig. 8G).

## Discussion

Melanoma is a relatively rare cancer, arising from the skin (88%), eye (9%), mucous membranes (1%), and unknown primary sites (2%) [31]. UVM is the most common primary intraocular malignant tumor in adults, representing approximately 85% of ocular melanoma [32]. The liver is the most commonly affected organ by metastatic UVM, with approximately 90% of patients demonstrating liver metastases at the time of death [33]. Despite successful local treatment of the primary tumor in the early stages, up to half of UVM patients eventually develop metastatic disease. This implies that UVM may already have microscopic metastases at the time of tumor onset. Once these micro-metastases become apparent, the prognosis is often poor, with disease-related death occurring within a year [34, 35].

To achieve personalized treatment, it is essential to understand the mechanisms of metastasis and the probability of metastases occurring. In this study, we used scRNA-seq data to investigate the key macrophage genes related to metastatic protective macrophages. We identified key genes related to macrophages in primary and metastatic samples using a high-dimensional weighted gene co-expression network analysis (HdWGCNA) method, and performed functional analysis and gene network analysis to deeply understand their biological functions and signaling pathways. Furthermore, based on the expression spectra of the key genes, we built machine learning and deep learning models using support vector machines and convolutional neural networks to differentiate primary patients from metastatic patients. In addition, we identified and characterized a prognostic model based on macrophage cell subtype key genes in UVM.

Through scRNA-seq analysis, we characterized the unique immune microenvironment landscape in UVM patients. We observed that macrophage cell abundance was higher in the primary group than in the metastatic group, suggesting the potential role of macrophages in the pathological biology of primary patients. Moreover, cluster analysis revealed differences in macrophage subpopulations between the two groups, with clusters 2, 4, 8, and 10 showing prominent activity in the primary group. By using HdWGCNA, we identified key genes related to macrophages in the primary group. The turquoise and brown modules were highly activated in clusters 2, 4, 8, and 10, suggesting their importance in primary macrophages. Functional analysis and gene network analysis of the top 80 genes in these modules revealed their involvement in antigen processing and presentation, immune effector processes, inflammatory response, and activation of immune response, indicating their importance in the immune response against tumor cells. Previous studies have often considered the role of the inflammatory microenvironment in UVM as a double-edged sword [36–38]. Indeed, our research has also found that antigen presentation and secretion of inflammatory factors coexisted in certain subgroups of macrophages. Therefore, HdWGNCA has provided us with a good means to decipher the inhibitory role of macrophages by identifying macrophages with strong antigen presentation capabilities in the microenvironment that protect against metastasis.

To further explore the potential clinical significance of the identified MPMφ, we used LASSO analysis to screen out five MPMRGs, and evaluated their ability to distinguish primary and metastatic groups using seven different machine learning models on the GSE22138 dataset. We then chose the more accurate SVM model and validated it on an independent external validation dataset GSE58294, showing a significant prediction effect (AUC = 0.813). Convolutional Neural Network (CNN) Deep Learning models are a type of deep learning model that utilizes convolutional learning for image recognition and have been applied in many studies with good predictive effects [39, 40]. We generated a heatmap for each patient in the training group and validation group, and using CNN to obtain the predicted AUC for the training group and validation group, which were 0.871 and 0.798, respectively. These findings provided a scheme and insight for predicting whether UVM tumors have metastasized. Our findings hold significant value for clinical practice because current clinical assessments of UVM patients for metastasis mainly rely on techniques like Positron Emission Tomography-Computed Tomograph (PET-CT), which may not easily detect occult lesions [41, 42]. However, our research methodology offers an efficient means to determine whether patients have metastasized.

Additionally, we established a UVM-macrophages related prognostic model based on five metastasis-related key macrophage genes (MPMRGs: ICAM1, FOS, PDE4B, CPVL, CD83). This model showed good overall survival predictive performance in both the TCGA-UVM cohort and an independent external validation cohort GSE84976(*p* < 0.0001), indicating its potential clinical application value. In a study of 500 cases of UVM, 48% of patients had 3p deletion, 27% had partial monosomy, and 25% had 3p complete monosomy. The 3-year cumulative metastasis rates for patients with 3p deletion, partial monosomy, and 3p complete monosomy were 3%, 5%, and 24%, respectively. The status of chromosome 3 is closely related to the prognosis of UVM, and patients with complete monosomy of chromosome 3 have the worst prognosis [43]. By using MPMRGs to perform risk scoring on GSE84976, we found that the high-risk group was predominantly composed of patients with complete monosomy of chromosome 3, while the low-risk group consisted mainly of patients with chromosome 3 double copies. This further demonstrates that risk scoring based on MPMRGs has better clinical significance. Among MPMRGs, we identified PDE4B as a independent protective genes PDE4B, a member of the phosphodiesterase superfamily, regulates intracellular cAMP levels by hydrolyzing cAMP to its inactive form [44]. Dysregulation of PDE4B has been implicated in various cancers [45, 46]. However, the role of PDE4B in UVM remains unknown. Mechanistically, macrophage PDE4B might contribute to reprogram TAM phenotype towards a pro-inflammatory state, offering therapeutic avenues for cancer intervention.

In summary, our study identified metastatic protective macrophages in UVM, which functionally related with antigen processing and inflammatory. These findings provided valuable insights into potential molecular mechanisms and the immune microenvironment of UVM metastasis and emphasized the importance of macrophages in the immune treatment response of lung cancer. Machine learning and deep learning models based on macrophage cell genes aid in differentiating tumor metastatic potential, and prognostic models based on macrophage cell genes have the potential to be prediction tools for patient outcomes, and may help in selecting appropriate immunotherapy strategies.

### Electronic supplementary material

Below is the link to the electronic supplementary material.

Supplementary Material 1

Supplementary Material 2

Supplementary Material 3

Supplementary Material 4

Supplementary Material 5

Supplementary Material 6

Supplementary Material 7

## Data Availability

The raw data of this article will be are available from the corresponding author upon reasonable request.

## Abbreviations

UVM: Uveal melanoma
HdWGCNA: High-dimensional weighted gene co-expression network analysis
MPMφ: Metastatic protective macrophages
NMF: Non-negative matrix factorization
MPMRGs: Metastatic protective macrophages related genes
CNN: Convolutional neural network
SEER: Surveillance, Epidemiology, and End Results
RR: Relative risk
TNF: Tumor necrosis factor
iNOS: Inducible nitric oxide synthase
ARG1: Arginase-1
MPMRGs: Macrophages related genes
DEGs: Differentially Expressed Genes
SVM: Support Vector Machine algorithm
