# Supplementary figures and images for: Single-cell hdWGCNA reveals metastatic protective macrophages and development of deep learning model in uveal melanoma
Source: J Transl Med. 2024 Jul 29;22:695. doi: 10.1186/s12967-024-05421-2 (PMC11287857; doi:10.1186/s12967-024-05421-2)

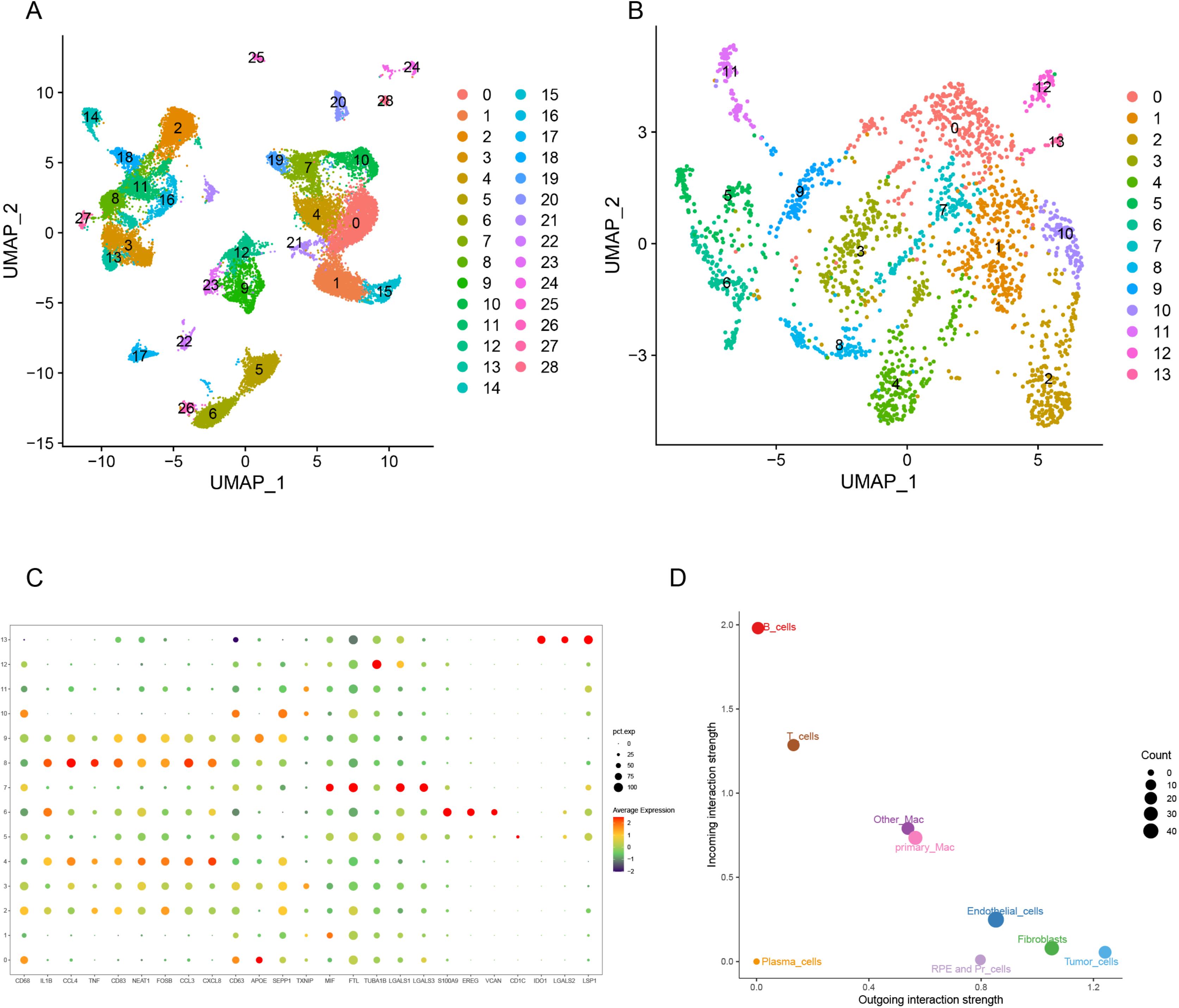

Supplement: Supplementary file 1 — Supplementary Material 1 [file 12967_2024_5421_MOESM1_ESM.jpg]

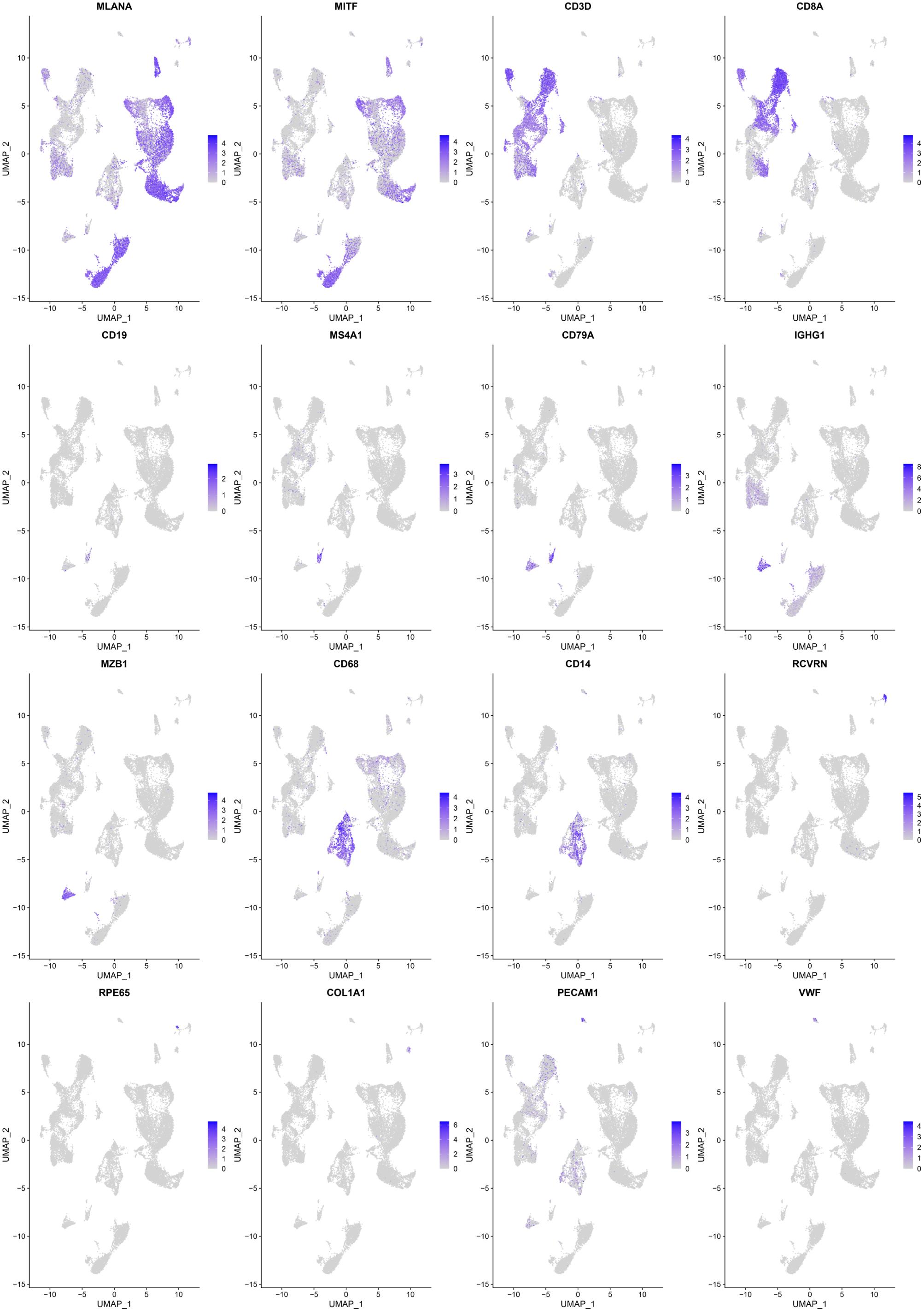

Supplement: Supplementary file 2 — Supplementary Material 2 [file 12967_2024_5421_MOESM2_ESM.jpg]
